# Selected nutritional habits of teenagers associated with overweight and obesity

**DOI:** 10.7717/peerj.3681

**Published:** 2017-09-22

**Authors:** Magdalena Zalewska, Elżbieta Maciorkowska

**Affiliations:** 1 Department of Public Health, Medical University of Bialystok, Białystok, Poland; 2 Department of Developmental Medicine and Pediatric Nursing, Medical University of Bialystok, Białystok, Poland

**Keywords:** Nutritional habits, Diet, Youth, Overweight and obesity

## Abstract

**Introduction:**

A balanced diet is at the heart of healthy growth and development of children and youth, whereas inappropriate eating habits considerably influence the incidence of disorders connected with nutrition, including overweight and obesity. This paper aims at studying nutritional factors of 18-year-old secondary school youth in the urban environment and their effect on the incidence of overweight and obesity.

**Materials and methods:**

The survey was conducted among 1,999 secondary school students chosen at random. The research tool consisted an original survey questionnaire. The measurements of respondents’ height and body mass provided data for calculating the body mass index.

**Results and conclusion:**

The percentage of youth with deficient body mass was estimated at 8.4%. The percentage of normal weight students in the surveyed group was estimated at 77.6%. Overweight and obesity characterized 14.0% of the total number. As many as 21.8% of overweight and obese respondents would eat one or two meals as opposed to 16.8% of normal weight students. Three-fourths of the surveyed students would eat breakfast regardless of their nutritional habits. Lunch is eaten by 52.9% of normal weight 18-year-olds and 46.1% of overweight and obese students. The analysis of mealtimes suggests that overweight and obese students would have their breakfast and dinner at later hours than the rest of the surveyed. More than half of the participating students failed to eat lunch (53.9%), and one in four students within this group resigned from supper. Girls would eat fruit and vegetables more frequently than boys several times a day. The percentage of persons in the surveyed groups who would eat fast foods on a daily basis was similar regardless of their nutritional status. Sweetened carbonated beverages would be drunk more often by overweight and obese boys (81.2%) as compared with boys with proper body mass (75.8%). The same type of beverages would be popular with two-thirds of girls, and this result was similar regardless of their nutritional status. About 44.2% of overweight and obese girls and 20% of girls with proper body mass attempted to lose weight, and 5.7% of boys tried to go on a diet. Eating limitations were declared by 16.5% of overweight and obese boys and ca. 3% of normal weight boys.

**Conclusion:**

Within the surveyed group of youth, it was possible to indicate eating errors primarily consisting in irregular eating, too low a number of meals during the day, particularly skipping breakfast, which took place more frequently among overweight and obese students rather than normal weight ones. The survey points to the insufficient intake of vegetables and fruit as opposed to salty and sweet meals. It is essential to convey the knowledge on the causes of overweight and obesity as well as rules of a healthy diet as factors preventing civilization diseases.

## Introduction

Excessive body mass is a common developmental disorder in the youth population of industrialized countries, and the popularity of their incidence tends to grow significantly and constitutes a serious problem of public health (Reilly & Kelly, 2011; Yanovski, 2015). Overweight and obesity in the pediatric population has attracted much attention because childhood and adolescence are critical developmental periods during which individuals establish the foundations for their future health (Sabo et al., 2012). Adolescence is a “high-risk period” for weight gain, characterized by critical changes in body composition, insulin sensitivity, eating and activity behaviors, and psychological adjustments (Alberga et al., 2012).

A balanced diet provides a basis for healthy growth and development of children and youth. In terms of eating, the kind of products, the method of their preparation, the number of meals and the length of intervals between them appears to be of importance. The basic factors contributing to the incidence of overweight and obesity are increased intake of high-energy foods, foods poor in nutrients and rich in fats, sugar and salt, as well as insufficient physical activity, which altogether lead to energetic imbalance of the whole organism (Leech, McNaughton & Timperio, 2014; Enes & Slater, 2010; Hill, Wyatt & Peters, 2012). Eating habits shaped throughout the childhood and strengthened during adult life are difficult to reshape. Improper diet of the development-age population may both cause developmental threats and irregular development in other, especially emotional and social, spheres of life (Sahoo et al., 2015; Todd et al., 2015; Popkin, Adair & Ng, 2012; Ng et al., 2014). Understanding the relationship between the nutritional condition and lifestyle, including nutritional habits, is necessary for effective prophylaxis and treatment of youth obesity.

The paper focuses on the study of nutritional behavior among 18-year-old secondary school youth of the urban environment as a factor affecting the incidence of overweight and obesity in the surveyed group.

## Materials and Methods

The survey was conducted among 1,999 18-year-old secondary school students in the months of September–October, 2011. Girls comprised 65.2% and boys 34.8% of the subjects. The research was conducted with randomly chosen classes in high schools (only governmental) in Bialystok city. We observed the predominance of girls in relation to boys (2:1) at schools (Central Statistical Office, 2016). Due to the strict chronological age-exclusion criterion and drop-off rate (invalid questionnaire response), participants were qualified for further analyses. Both participants and school authorities gave their informed written consent for the study and approved the protocol.

Pilot studies were conducted on 30 students for the validation and introduction of modification of a questionnaire. Analysis of eating habits included: number of meals, time and place of meals and snacking, presence of sugar and salt in the diet, also the impact of stressful situations (such as tests at school, school failure, relations with parents, lack of acceptance among the peers and appearance) on appetite (bigger, unchanged or poor). For an assessment of the intake of the selected products (bread, dairy, meat, fish, sweet, fruit, vegetable and fast food) a scale was used: rarely or never, several times a week (two to seven times), several times a day (two times or more). Body weight and height were measured using an electronic scale (Seca) and Holtain anthropometer. Body mass index (BMI) was calculated for each individual in a standard way in accordance with the World Health Organization (WHO) criteria. BMI < 5 percentile indicated thinness, and BMI ≥ 85 overweight and obesity. BMI values in between 5 and 85 percentile referred to normal weight youth. For further analysis of the nutritional status and dietary behaviors, BMI values were recorded for normal weight and overweight and obesity students only (1,832 participants) excluding BMI < 5 percentile.

The statistical analysis of the obtained results was conducted by using a statistical package Statistica PL 8.0. The analysis required the application of the test for independence χ^2^. In addition, the post hoc pairwise comparisons test including Bonferroni correction for multiple comparison was used. The occurrence of differences of statistical significance was analyzed at the significance level of *p* < 0.05. The survey obtained consent from the Bioethics Committee of the Medical University of Bialystok (Ref.: R-I-002/74/2011).

## Results

The percentage of normal weight students comprised 77.6%, girls—78.2% and boys—76.6%. The survey indicated thinness with 8.4% of the surveyed students (10.5% of girls and 4.3% of boys). About 9.4% of respondents were overweight (8.5% of girls and 11.1% of boys), whereas 4.6% of the surveyed students were obese (2.8% of girls and 8.0% of boys).

Overweight and obese students would have fewer meals during the course of the day as compared with other groups. As many as 21.8% of overweight and obese students (24.5% of girls and 18.8% of boys) would eat one or two meals a day, unlike 16.8% of normal weight students (18.8% of girls and 12.8% of boys). A smaller number of students with overweight and obesity (78.2%) would have three meals during the day in comparison with normal weight students (83.2%) (Table 1).

**Table 1:** Eating of meals in the surveyed group of youth.

Analyzed factor		5–85 BMI percentile		≥85 BMI percentile		p*
		*n*	%	*n*	%	
Girls	<3 meals	18.8	192	24.5	36	0.107
	≥3 meals	81.2	827	75.5	111	
Boys	<3 meals	12.8	68	18.8	25	0.072
	≥3 meals	87.2	465	81.2	108	
Total	<3 meals	16.8*	260	21.8*	61	0.041*
	≥3 meals	83.2*	1,292	78.2*	219	

**Note:**

* Pearson’s chi-squared test.

Three fourths of the surveyed students would eat breakfast. The surveyed girls regardless of their nutritional status would eat breakfast at a similar percentage. However, there are discrepancies in terms of overweight and obese (75.2%) and normal weight boys (81.8%) (*p* = 0.085). Lunch is eaten by more normal weight 18-year-olds (52.9%) as compared with the group of overweight and obese students (46.1%) (*p* < 0.05). Boys with a normal BMI who would eat their lunch constituted 50.3%, whereas those that were overweight and obese—43.6%. Boys that were overweight and obese would have their breakfast at a later time than the other groups. The youth who would have dinner between 2:00 PM and 5:00 PM constituted 61.0%. About 15.6% of the normal weight and one in four obese students admitted having dinner after 5:00 PM. More than half of the overweight and obese respondents skipped lunch (53.9%) and almost one in four of them resigned from supper. This might indicate that youngsters characterized by being overweight and obese accumulated meals and ate the bulk part of their daily ration in the afternoon (Table 2).

**Table 2:** Usual mealtimes among the surveyed group of youth.

Mealtimes		5–85 BMI percentile		≥85 BMI percentile		p*	p**
		*n*	%	*n*	%		5–85 BMI percentile vs ≥85 BMI percentile
Breakfast	<8 AM	924	59.6	136	48.6	0.001	<0.017
	≥8 AM	300	19.3	78	27.8		<0.017
	Skipped	328	21.1	66	23.6		ns
Lunch	<11 AM	225	14.5	32	11.4	0.092	ns
	≥11 AM	596	38.4	97	34.7		ns
	Skipped	731	47.1	151	53.9		<0.017
Dinner	<2 PM	210	13.5	35	12.5	0.003	ns
	2–5 PM	965	62.2	148	52.9		<0.013
	>5 PM	242	15.6	65	23.2		<0.013
	Skipped	135	8.7	32	11.4		ns
Supper	<8 PM	745	48.0	146	52.1	<0.001	ns
	≥8 PM	554	35.7	68	24.3		<0.016
	Skipped	253	16.3	66	23.6		<0.016

**Notes:**

* Pearson’s chi-squared test.

** Post hoc pairwise comparisons test including Bonferroni correction for multiple comparison.

Snacking between main meals was found with the majority of students (96.1% of girls and 94.1% of boys). A significant percentage, i.e., 33.1% of girls and 36.7% of boys declared snacking between meals several times a day. The most frequently eaten products were fruits (77.2%), sweets (64.1%), yoghurt (61.1%) and sandwiches (57.0%). Girls would prefer fruits (84.5%), sweets (67.5%), sandwiches (62.7%) and yoghurt (57.1%). Boys eagerly chose yoghurt (58.6%), sandwiches (56.9%) and sweet snacks (56.9%). Students with excessive body weight would regularly snack on fruits (78.3%), sweets (65.3%), sandwiches (54.7%) and yoghurts (60.3%). Boys would more often opt for these types of products than girls (these were eaten by 7.9% and 4.3%, respectively, on a daily basis). Merely 15.9% of the surveyed students resigned from fast foods (16.1% of girls and 15.4% of boys). The percentage of respondents turning to fast foods every day in the surveyed group was similar regardless of their nutritional status (5.5% of normal weight respondents and 4.6% of overweight and obese students). Fast food was chosen by every fifth student for snacking. About 79.5% of normal weight students and 73.6% of overweight and obese youngsters would eat fast food several times a month. It appears that one-third of overweight and obese girls would never snack on fast food products. Girls with proper body mass refraining from fast foods constituted 15.5%. Boys who would never eat fast foods made 14.1% of the normal weight and 19.5% of the overweight and obese respondents. Nearly half of the surveyed students (46.2%) declared salting their foodstuffs and dishes. Salting food before eating was more common with obese (57.6%) rather than normal weight (43.3%) students. Obese boys who salted their food products and meals constituted 65.5%, whereas obese girls—50.0%.

Normal weight youth who declared sweetening beverages, food products and dishes comprised 48.4%, whereas youth with overweight and obesity constituted the smallest percentage (40.7%). Boys more frequently admitted to sweetening their food (55.2%) rather than girls (45.2%). Sweetened carbonated beverages were more popular among boys (76.9%) than girls (69.3%). It is of note, however, that overweight and obese students more frequently declared drinking such beverages (81.2%) as opposed to boys with a normal body mass (75.8%). Drinking sweetened carbonated drinks was common among two-third of the girls and was similar regardless of their nutritional status. Almost all the surveyed students (95.9%) would eat sweets, of whom one fourth would do so on a daily basis (Table 3). Girls, more often than boys, declared eating sweet treats. Overweight and obese youth would less often eat sweets, like normal weight students.

**Table 3:** Eating sweets in the surveyed group of students.

Eating sweets		5–85 BMI percentile		≥85 BMI percentile		p*	p**
		*n*	%	*n*	%		5–85 BMI percentile vs ≥85 BMI percentile
Girls	Rarely or none	31	3.0	10	5.4	0.001	<0.016
	Several times a week	735	72.1	117	77.6		ns
	Several times a day	253	24.8	20	17.0		<0.016
Boys	Rarely or none	25	4.7	12	10.5	0.003	ns
	Several times a week	380	71.3	105	81.2		ns
	Several times a day	128	24.0	16	8.3		<0.016
Total	Rarely or none	56	3.6	22	7.9	<0.001	<0.016
	Several times a week	1115	71.8	222	79.3		<0.016
	Several times a day	381	24.5	36	12.8		<0.016

**Notes:**

* Pearson’s chi-squared test.

** Post hoc pairwise comparisons test including Bonferroni correction for multiple comparison.

The surveyed students who would eat vegetables several times a day constituted 42.8%. Girls would more often declare eating vegetables a few times a day than boys (45.9% and 36.9%, respectively). Lack of vegetables in a daily diet was confirmed by 11.3% of girls and 14.2% of boys, of whom 3.7% of girls and 7.2% of boys resigned from eating vegetables at all. More than half of the overweight and obese eat vegetables several times daily. Girls with proper body mass who would opt for vegetables a few times a day comprised 44.1%. A similar percentage (39.8% and 37%, respectively) of overweight and obese boys as well as boys with proper body mass would eat vegetables several times a day (Table 4).

**Table 4:** Eating vegetables in the surveyed group of students.

Eating vegetables		5–85 BMI percentile		≥85 BMI percentile		p*	p**
		*n*	%	*n*	%		5–85 BMI percentile vs ≥85 BMI percentile
Girls	Rarely or none	40	3.9	8	5.4	0.064	ns
	Several times a week	530	52.0	89	60.5		ns
	Several times a day	449	44.1	50	34.1		<0.016
Boys	Rarely or none	37	6.9	5	6.0	<0.001	ns
	Several times a week	299	56.1	45	54.2		<0.016
	Several times a day	197	37.0	83	39.8		<0.016
Total	Rarely or none	77	5.0	13	4.6	0.186	ns
	Several times a week	829	53.4	134	47.9		ns
	Several times a day	646	41.6	133	47.5		ns

**Notes:**

* Pearson’s chi-squared test.

** Post hoc pairwise comparisons test including Bonferroni correction for multiple comparison.

Eating fruits several times a day was declared by 46.3% of the surveyed, where girls (49.4%) opted for them more often than boys (40.4%). A similar percentage of overweight and obese as well as normal weight students confirmed eating several portions of fruits daily (Table 5).

**Table 5:** Eating fruits in the surveyed groups of students.

Eating fruits		5–85 BMI percentile		≥85 BMI percentile		p*	p**
		*n*	%	*n*	%		5–85 BMI percentile vs ≥85 BMI percentile
Girls	Rarely or none	32	3.1	6	4.1	0.695	ns
	Several times a week	496	48.7	67	45.6		ns
	Several times a day	491	48.2	74	50.3		ns
Boys	Rarely or none	41	7.7	5	3.8	0.259	ns
	Several times a week	278	52.1	70	52.6		ns
	Several times a day	214	40.2	58	43.6		ns
Total	Rarely or none	73	4.7	11	3.9	0.776	ns
	Several times a week	774	49.9	137	48.9		ns
	Several times a day	705	45.4	132	47.2		ns

**Notes:**

* Pearson’s chi-squared test.

** Post hoc pairwise comparisons test including Bonferroni correction for multiple comparison.

More than one-third of overweight and obese students admitted that they eat more than usual under stress. Normal weight students eating more in stressful situations comprised 28.1%. As many as 50% of girls with excessive body weight would eat more while under pressure, whereas girls with normal body weight amounted to 37.5%. Boys eating more in stressful situations constituted 19.2% in the group of overweight and obese respondents, 10.9% in the normal weight. By contrast, 33.5% of normal weight and 26.7% of overweight and obese 18-year-olds would eat smaller amounts of food. About 42.2% of overweight and obese girls stated that they attempted to lose weight. This was also done by every fifth girl with a proper body mass. Among boys, 5.7% confirmed having tried to go on a diet. About 16.5% of overweight and obese boys and 3% of boys strove to limit their food intake.

## Discussion

Our survey aimed at defining eating habits among youth of diversified nutritional status. It follows that eating regular meals facilitates proper body weight with youngsters. The surveyed students with overweight and obesity would eat fewer meals during a day than eutrophic students (one or two meals were eaten by 21.8% overweight and obese students and 16.8% of eutrophic school goers). The differences in the number of meals were particularly visible among girls. Obese girls would eat significantly fewer meals (one or two meals eaten by 24.2%) than girls with proper body mass (18.8% would eat one or two meals). Other research confirms that eating the same volume of food within just one or two meals (instead of recommended five meals) evokes faster deposition of adipose tissue, which contributes to the development of obesity (Sygit et al., 2012; Kulovitz et al., 2014; Bachman et al., 2011). The significance of breakfast in providing children and youth with adequate nutrients was documented in several studies (Szajewska & Ruszczynski, 2010; de la Hunty, Gibson & Ashwell, 2013; Leidy et al., 2013). The NHANES survey conducted among children and young adults aged 9–18 indicated that a considerable percentage of children and youth (20–30%) skipped breakfast, and this was more common in a group of older teenagers, especially girls (Deshmukh-Taskar et al., 2010). Our survey proves that skipping breakfast may correlate with increased risk of disorders in the nutritional status. Among the surveyed 18-year-olds, 80.1% of those with a proper BMI would eat breakfast, whereas the percentage was smaller with overweight/obese students (74.2%). It was proved that children and young adults who skip breakfast at home, compensate for that fact by eating even bigger amount of food as the day develops (Kral et al., 2011). Other studies (Tin et al., 2011; Fayet-Moore et al., 2016; Smith et al., 2010) indicate the existence of a relationship between having breakfast and BMI. Children skipping breakfast ran a higher risk of obesity as compared to those eating regular breakfast. The survey by Øverby & Høigaard (2012) conducted among Norwegian teenagers pointed to a low percentage of young people eating regular lunch—it was lower in the case of girls (50.6%) rather than boys (59.2%). Similarly in individual surveys, lunch was more frequently skipped by girls than boys. The percentage of 18-year-olds eating lunch constituted merely 46.6% among overweight and obese students, and it was the lowest in comparison to eutrophic. This may result from false conviction that skipping meals, breakfast in particular, facilitates body mass loss and is a frequent method of dieting.

The surveys conducted in the USA suggest that snacking comprises the main factor responsible for increasing total energy supply that has been observed for the last 30 years (Duffey & Popkin, 2011). The students in our survey admitted snacking in the following proportions: 96.1% of girls and 94.1% of boys. A significant percentage, i.e., 33.1% of girls and 36.7% of boys, declared snacking after school, while watching television and usually among their peers. Snacks between meals are popular with children and young adults, however, as the surveys show (Reicks et al., 2015; Chapelot, 2011), these are highly processed products, enriched with fat, sugar and salt. Above all, those who skip main meals eat less fruits and vegetables but more white bread, sweet beverages, and eat sweet snacks (Lazzeri et al., 2013).

Studies prove that children and youth, especially older boys, constitute the main consumers of fast foods. The consumption of this type of food may lead to vitamin deficiency, and ultimately to obesity which, in turn, causes many eating-dependent diseases. Moreover, children and youth eating this type of products show a tendency to eat a larger number of calories than those who resign from such food (Pangan et al., 2012; Hearst et al., 2013; Washi & Ageib, 2010; Poti, Duffey & Popkin, 2014). In our survey, a considerable percentage (84.1%) confirmed eating fast food, where one-fifth would eat it at least once a week. It is more popular among boys than girls. Among the surveyed students, there were persons (7.9% of girls and 4.3% of boys) who would opt for such products every day. The percentage of respondents eating fast foods on a daily basis in the surveyed group was similar regardless of their nutritional status.

In addition, eating fast food is frequently accompanied by drinking sweetened beverages, which increases with age and is more common with boys rather than girls (Currie et al., 2012; Mazur & Małkowska-Szkutnik, 2011). The analysis of data concerning eating habits among children and youth (4–18 years) in Great Britain indicated a significant interdependence between eating salt and total intake of beverages, mainly sweetened ones (He, Marrero & MacGregor, 2008). Our study indicates that sweetened carbonated drinks were popular with two-thirds of the surveyed students, boys in particular. Eating sweet snacks was more common among girls, whereas boys would rather choose sweetened drinks (Vanderlee et al., 2014). Similarly, in our study, more girls than boys declared eating sweet snacks. Overweight and obese students would less often turn to sweets rather than normal weight respondents. The WHO recommends a diet poor in fat, sugar and salt, whereas rich in fruits and vegetables (World Health Organization, 2004). In the HBSC 2009–2010 survey, merely 33% of girls and 25% of boys at the age of 15 stated that they eat fruits a minimum of once a day. Similar results concerned eating vegetables (only one-third of the surveyed youth) (Currie et al., 2012). Girls, more often than boys, declared eating fruits and vegetables (Currie et al., 2012; Iannotti & Wang, 2013), which corresponds to our individual survey. Adolescence is a period of change in body size, shape and composition during the pubertal period, which may trigger body dissatisfaction and unhealthy eating and weight control practices, such as skipping meals, severely restricting intake of carbohydrate, protein or dairy foods, laxative use and smoking (Todd et al., 2015). The Polish survey of young adults of various nutritional statuses indicates that overweight and obese youngsters undertake actions to lose weight consisting in eating a smaller number of meals a day, skipping breakfast, avoiding sweet and salty snacks, as well as increasing fruits and vegetables intake (Jodkowska et al., 2011). Our study shows that almost 50% of overweight and obese girls as well as one in five girls with a normal body mass declared dieting. Among boys, 5.7% confirmed having attempted to lose weight, mostly overweight and obese boys (16.5%).

The development of overweight and obesity may result from stress connected with unhealthy eating habits. Stress-related eating behavior was more common among girls (43%) rather than boys (15%), and persons eating more in stressful situations were characterized by a higher incidence of overweight and obesity (Jääskeläinen et al., 2014). In our research on students with excessive body weight, as many as 50% of girls and almost 20% of boys would eat more in stress-related situations. Other studies suggest a relationship between feeling stressed and eating sweet and fatty dishes, and a negative relationship toward eating fruits and vegetables (Michels et al., 2012). The results of our survey confirm errors in the mode of eating among young adults. Nutritional habits disadvantageous to health should be revised through activities connected with dietary education. The youth should be aware of complex mechanisms of arising nutritional disorders, capable of identifying their symptoms and be conscious of health-related effects stemming from erroneous eating habits.

## Limitations

There were some limitations to our study, however. First, it was possible to gather data on eating behavior and its background only by using the results of a survey. We did not take into account the impact of socioeconomic factors on students’ eating habits. The study was conducted among governmental schools only. Multicenter studies should be performed to confirm whether there is an addition association between BMI and selected eating habits. Additionally, the conducted study accounted for anthropometric measurements (of body mass and height) and led to the creation of three categories of children characterized by different nutritional statuses (thinness, normal weight, overweight and obese). Overweight and obese youngsters could already have developed certain habits connected with dieting and limiting their intake of selected products. Nevertheless, it is an interesting issue for further studies concerning nutritional habits among young people. This study remains a crucial source of information, providing a basis for developing directions of future prophylactic undertakings.

## Conclusion

Our research indicates that obese youngsters tried to control the caloric intake through limiting the number of meals. The importance of breakfast in maintaining proper body mass among children and young adults was confirmed in various studies. In the surveyed group of youngsters, it was possible to indicate eating errors primarily consisting in irregular mealtimes, too low a number of meals during a day, especially skipping breakfast, which would take place more frequently with overweight and obese students rather than normal weight respondents. The survey points to insufficient intake of vegetables and fruits, but high intake of salty and sweet dishes in the surveyed group of students. It is necessary to convey the knowledge on the causes of overweight and obesity and the rules of healthy dieting as factors for preventing civilization diseases.

## Additional Information and Declarations

### Data Availability

The following information was supplied regarding data availability: The raw data are included in the tables as percentage representation of coded values.
